# Human cytomegalovirus transcriptome activity differs during replication in human fibroblast, epithelial and astrocyte cell lines

**DOI:** 10.1099/vir.0.038083-0

**Published:** 2012-05

**Authors:** James C. Towler, Bahram Ebrahimi, Brian Lane, Andrew J. Davison, Derrick J. Dargan

**Affiliations:** 1 MRC – University of Glasgow Centre for Virus Research, Glasgow G11 5JR, UK; 2 Department of Functional and Comparative Genomics, Institute of Integrative Biology, University of Liverpool, Biosciences Building, Crown Street, Liverpool L69 7ZB, UK; 3 Liverpool Microarray Facility, Institute of Integrative Biology, University of Liverpool, Biosciences Building, Crown Street, Liverpool L69 7ZB, UK

## Abstract

Broad cell tropism contributes to the pathogenesis of human cytomegalovirus (HCMV), but the extent to which cell type influences HCMV gene expression is unclear. A bespoke HCMV DNA microarray was used to monitor the transcriptome activity of the low passage Merlin strain of HCMV at 12, 24, 48 and 72 h post-infection, during a single round of replication in human fetal foreskin fibroblast cells (HFFF-2s), human retinal pigmented epithelial cells (RPE-1s) and human astrocytoma cells (U373MGs). In order to correlate transcriptome activity with concurrent biological responses, viral cytopathic effect, growth kinetics and genomic loads were examined in the three cell types. The temporal expression pattern of viral genes was broadly similar in HFFF-2s and RPE-1s, but dramatically different in U373MGs. Of the 165 known HCMV protein-coding genes, 41 and 48 were differentially regulated in RPE-1s and U373MGs, respectively, compared with HFFF-2s, and 22 of these were differentially regulated in both RPE-1s and U373MGs. In RPE-1s, all differentially regulated genes were downregulated, but, in U373MGs, some were down- and others upregulated. Differentially regulated genes were identified among the immediate-early, early, early late and true-late viral gene classes. Grouping of downregulated genes according to function at landmark stages of the replication cycle led to the identification of potential bottleneck stages (genome replication, virion assembly, and virion maturation and release) that may account for cell type-dependent viral growth kinetics. The possibility that cell type-specific differences in expressed cellular factors are responsible for modulation of viral gene expression is discussed.

## Introduction

Human cytomegalovirus (HCMV; species *Human herpesvirus 5*) is typically acquired in early childhood, although in developed countries this is often delayed. The virus establishes a life-long, persistent infection in myeloid progenitor cells (Kondo *et al.*, 1994). The symptoms of primary infection in healthy adults are generally mild, but infection during pregnancy can result in severe neurological damage to the fetus. HCMV is also a major contributor to morbidity and mortality among immunocompromised individuals. During the acute phase of primary infection, most body organs and a wide variety of cell types are infected (Sinzger *et al.*, 1995). Indeed, the broad host cell tropism exhibited by HCMV *in vivo* is an important factor in its pathogenesis. However, the extent to which HCMV gene expression at the transcriptional level, and hence viral replication, is modulated because of the involvement of different cell types is unknown. In HCMV-permissive cell culture systems, both cytopathic effect (CPE) and infectious viral yield can vary markedly between cell types (Wang *et al.*, 2008).

The HCMV genome is organized into unique long and unique short regions (U_L_ and U_S_), each bounded by terminal and internal inverted repeats (TR_L_/IR_L_ and TR_S_/IR_S_). The genome of the low passage strain Merlin is 235 646 bp in size and contains the estimated 165 protein-coding ORFs that are present in wild-type strains, although two (RL13 and UL128) are interrupted by point mutations as a result of their instability in fibroblast cell culture (Dargan *et al.*, 2010; Davison *et al.*, 2003; Dolan *et al.*, 2004; Stanton *et al.*, 2010). Many HCMV genes are dispensable for growth in cell culture, and provide functions involved in processes such as cell tropism, immune evasion and viral temperance (suppression of viral growth) (Davison & Bhella, 2007).

Regulation of HCMV gene expression is complex, and offers the potential for cell type-dependent regulation. Viral genes are transcribed by host RNA polymerase II in a regulated temporal cascade of immediate-early (IE), early (E), early late (E-L) and late (L) genes. Temporal regulation of some genes (e.g. UL4 and UL44) is mediated by alternative promoters at different stages in the replication cycle (Isomura *et al.*, 2007; Kerry *et al.*, 1996, 1997). Transcripts that are antisense to viral mRNAs are produced in infected fibroblast and endothelial cells, and might have regulatory roles (Gatherer *et al.*, 2011; Zhang *et al.*, 2007). HCMV also encodes several miRNAs (Grey & Nelson, 2008), some of which may participate in control of viral or cellular gene expression (Stern-Ginossar *et al.*, 2007). The HCMV genome contains numerous binding sites for cellular transcription factors, and these may promote cell type-dependent differences in viral transcription kinetics and thus influence viral replication and pathogenesis.

We investigated HCMV replication kinetics and synthesis of viral DNA and RNA in human fetal foreskin fibroblast cells (HFFF-2s), retinal pigmented epithelial cells (RPE-1s; Miceli *et al.*, 1989) and astrocytoma cells (U373MGs; Kari *et al.*, 1992), as representative of cell types that are infected *in vivo*. Transcriptome profiling using DNA microarrays has historically provided a powerful technique for rapid analysis of global viral gene expression, and can help identify key regulatory effects above and beyond those due to gene-specific mechanisms. This technology has been employed for HCMV (Chambers *et al.*, 1999; Yang *et al.*, 2006) and several other herpesviruses (Aguilar *et al.*, 2005; Ebrahimi *et al.*, 2003; Jenner *et al.*, 2001; Kennedy *et al.*, 2005; Li *et al.*, 2006; Ohyashiki *et al.*, 2005; Stingley *et al.*, 2000; Tang *et al.*, 2006; Tsao *et al.*, 2009). Each of these studies investigated viral transcriptome activity in a single cell line, except for Kennedy *et al.* (2005), which found evidence for differential expression of varicella-zoster virus genes in two cell lines. We have developed a bespoke HCMV microarray platform to investigate transcriptome activity in three different cell types during a single round of replication by strain Merlin. We found that downregulation of certain virus genes may cause bottlenecks that operate at landmark stages in the HCMV replication cycle.

## Results

In order to correlate viral transcriptome activity in HFFF-2s, RPE-1s and U373MGs with concurrent biological responses, we examined viral CPE, growth kinetics and genomic loads.

### Viral growth kinetics

**Fig. 1.**
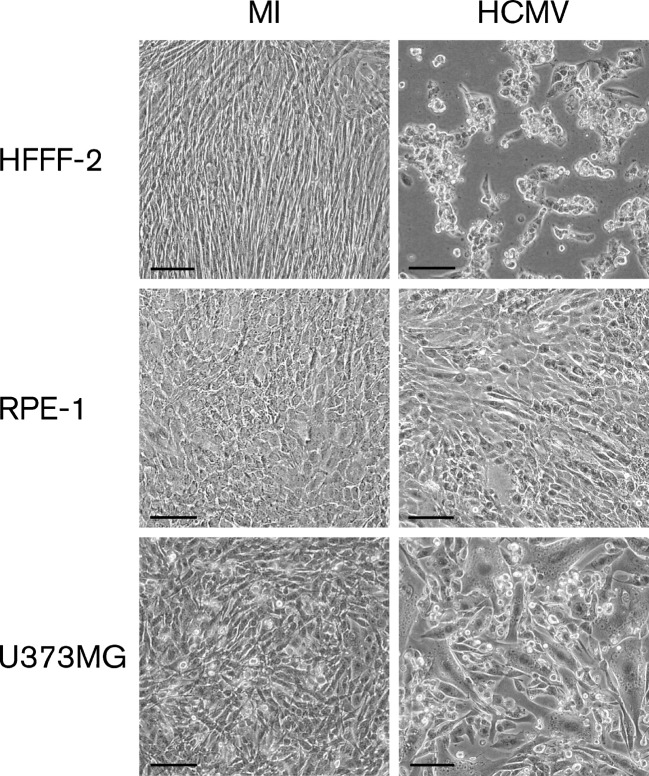
HCMV CPE at 72 h p.i. in HFFF-2s, RPE-1s and U373MGs infected at 6 p.f.u. per cell. MI, Mock-infected cells; HCMV, HCMV-infected cells. Bar, 500 µm.

The ability of strain Merlin to grow in HFFF-2s, RPE-1s and U373MGs after infection at high m.o.i. values was investigated. The extent of CPE at 72 h post-infection (p.i.) depended on the cell type (Fig. 1). All cells in the HFFF-2 monolayers were rounded and clumped. In contrast, the U373MG and RPE-1 monolayers stayed intact, with cells remaining in contact with each other. Swollen and syncytial cells were a common feature of infected U373MG monolayers, but were less frequent in RPE-1 monolayers, which exhibited little evidence of CPE. Thus, the cell type-dependent CPE exhibited by strain Merlin is similar to that reported previously for strains AD169 and Towne in primary RPEs and astrocyte cultures (Detrick *et al.*, 1996; Poland *et al.*, 1990).

**Fig. 2.**
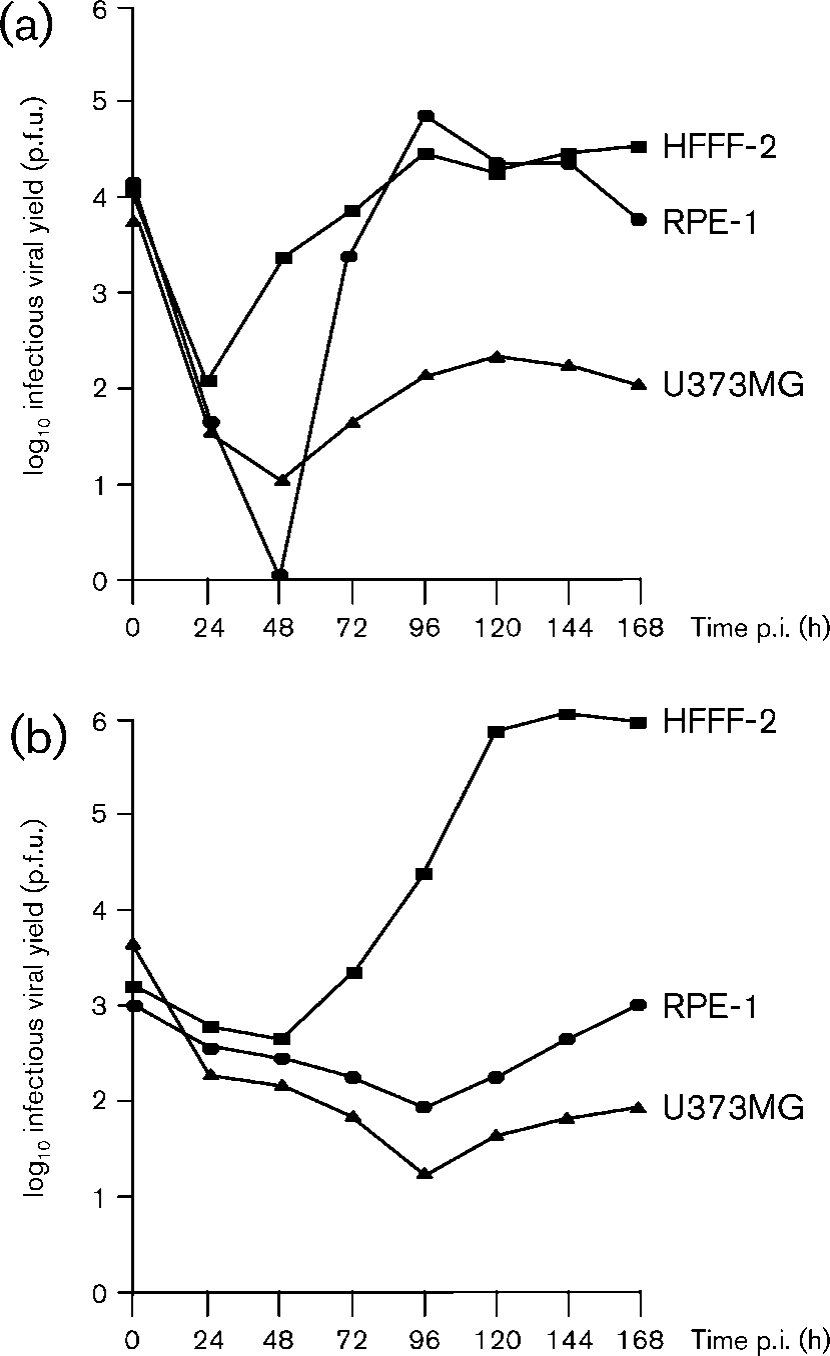
Single-step growth curves of HCMV in HFFF-2s, RPE-1s and U373MGs infected at 6 p.f.u. per cell: (a) CAV and (b) CRV.

Viral growth kinetics were compared in single-step growth experiments performed simultaneously with the three cell lines. The data were obtained from a single experiment, performed at the same time as the infections for microarray analysis (see below), using the same viral stock and batches of cells. Infectious viral yields from cells infected at an m.o.i. of 6 p.f.u. per cell were separated into cell-associated virus (CAV; Fig. 2a) and cell-released virus (CRV; Fig. 2b), and titrated on HFFF-2s. Compared with HFFF-2s, exit from the eclipse phase was delayed by 24 h in RPE-1s and U373MGs (Fig. 2a). Peak CAV titres were similar in HFFF-2s and RPE-1s, but were approximately 100-fold lower in U373MGs, and CAV accumulated most rapidly in RPE-1s and most slowly in U373MGs (Fig. 2a). CRV production was first detected at 72 h p.i. in HFFF-2s, but not until 120 h p.i. in RPE-1s and U373MGs, and final yields were 1000- and 10 000-fold lower, respectively, than in HFFF-2s (Fig. 2b). These results show that strain Merlin exhibits cell type-dependent growth properties similar to those of other strains (Detrick *et al.*, 1996; Poland *et al.*, 1990).

To determine whether the three cell lines differ in susceptibility to viral infection, monolayers were infected with strain Merlin at 6 p.f.u. per cell and the numbers of infected cells were estimated by immunofluorescent detection of the UL44 protein. Compared with HFFF-2s (100 %), 82 % of RPE-1s and 77 % of U373MGs were identified as infected. These small differences cannot by themselves account for the observed cell type-specific differences in viral growth.

### Viral DNA yield

Whole cell DNA was harvested from HFFF-2s, RPE-1s and U373MGs either mock-infected (MI) or infected with HCMV at 6 p.f.u. per cell. Viral DNA yield was determined by real-time quantitative PCR (qPCR), and increased at a much slower rate in RPE-1s and U373MGs than HFFF-2s (Table 1). At 96 h p.i., when infectious viral yield approached peak levels in each cell line (Fig. 2a), the viral DNA yield in HFFF-2s was 48- and 129-fold greater than in RPE-1s and U373MGs, respectively. It might be expected that the relatively low levels of HCMV DNA replication in RPE-1s and U373MGs (Table 1) contribute to delayed viral replication kinetics and lower viral yield (Fig. 2). However, a direct correlation cannot be drawn because the data in Table 1 represent total viral DNA, whether packaged or unpackaged. This suggested that cell type-dependent differences in viral maturation were likely to be important.

**Table 1.** Estimates of HCMV genome loads Single experiment. Standard curve: slope = −3.22; *R*^2^ = 0.965. nd, Not done.

Cell type	Stage (h p.i.)	Load (genomes per cell)
HFFF-2	MI	<0.06
	24	220
	48	7 900
	72	31 000
	96	62 000
RPE-1	MI	<0.07
	24	12
	48	nd
	72	270
	96	1 300
U373MG	MI	<0.07
	24	170
	48	250
	72	300
	96	480

### Viral transcriptome activity

**Fig. 3.**
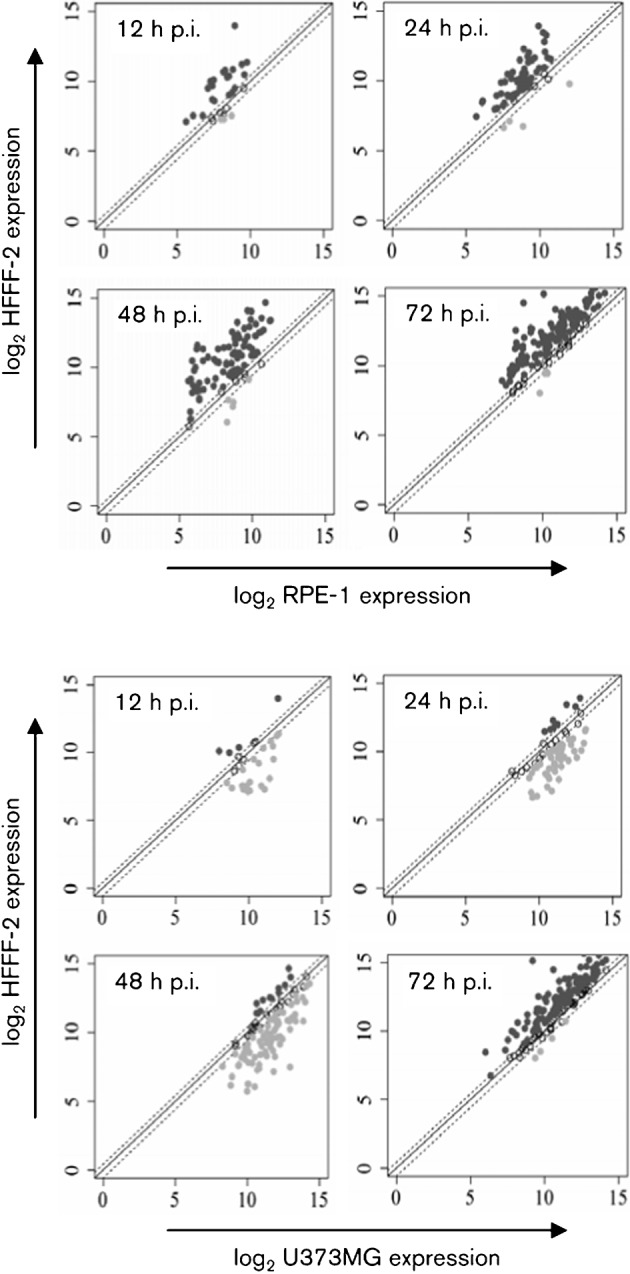
Scatter plots showing time-specific comparisons of HCMV gene expression in RPE-1s and U373MGs relative to HFFF-2s. The solid line denotes equivalent expression levels and the broken lines indicate±twofold differences, and dark and light grey points represent ORFs outside these boundaries.

A microarray platform specific for strain Merlin was developed to examine cell type-dependent differences in viral gene expression (i.e. transcript abundance) among HFFF-2s, RPE-1s and U373MGs. The data are presented in full in Table S1 (available in JGV Online). Expression of approximately 96 % of the total number of viral ORFs was detected in HFFF-2s and RPE-1s, and 98 % in U373MGs. Most ORFs were expressed at similar levels in HFFF-2s and RPE-1s (Fig. 3 and Table S1), with a temporally ordered increase in the numbers expressed. However, the pattern of expression in U373MGs was markedly different, with most ORFs, including those from genes exhibiting L kinetics in fibroblasts, expressed as early as 24 h p.i. (Table S1). Thus, compared with HFFF-2s and RPE-1s, the HCMV transcription cascade in U373MGs was either completed much earlier or significantly deregulated.

Transcripts from a greater number of ORFs were detected at earlier time points in RPE-1s and U373MGs than in HFFF-2s, although those from a small number were first detected in HFFF-2s (Table 2 and Table S1). Totals of 41 and 48 ORFs were differentially expressed (*P*<0.05) in RPE-1s and U373MGs (Table 2), respectively, compared with HFFF-2s. Of these, 13/41 ORFs in RPE-1s and 7/48 ORFs in U373MG were differentially expressed at multiple time points. In RPE-1s, all differentially expressed ORFs were expressed at lower levels than in HFFF-2s. However, in U373MGs, some ORFs were expressed at lower and others at higher levels compared with HFFF-2s, and a few exhibited a biphasic response (decreased at one time point and increased at another) (Table 2). Scrutiny of Table 2 reveals that 22 ORFs were differentially regulated in both RPE-1s and U373MGs.

**Table 2.** Differential expression of HCMV genes in RPE-1s and U373MGs compared with HFFF-2s Values are FCs in viral ORF signal intensity in RPE-1s/U373MGs relative to HFFF-2s: +, upregulated; −, downregulated. Only ORFs showing FCs>2 and *P*<0.05 are listed. nsd, No significant difference in signal between HFFF-2s and RPE-1s/U373MGs; sd, small difference in FC (<2). Selected 3′-co-terminal genes are in bold type. Protein properties and functions were adapted from Davison & Bhella (2007) by permission of Cambridge University Press.

Gene	Class	12 h p.i.	24 h p.i.	48 h p.i.	72 h p.i.	Protein properties and functions
**RPE-1**						
RL5A		*	†	−7.3	nsd	RL11 family
RL10	E-L	*	*	−3.4	nsd	Virion envelope glycoprotein
RL11		*	nsd	−21.9	nsd	RL11 family; IgG Fc-binding membrane glycoprotein
RL12		*	nsd	−7.7	nsd	RL11 family; putative membrane glycoprotein
RL13	E-L	*	nsd	−17.3	nsd	RL11 family, virion envelope glycoprotein
**UL4**	E-L	−5.8	−9.9	−14	nsd	RL11 family; virion envelope glycoprotein
**UL5**	E-L	nsd	−6.0	−4.1	nsd	RL11 family; putative membrane protein
UL10	E-L	*	*	−15.2	nsd	RL11 family; putative membrane glycoprotein
UL11	E	‡	nsd	−26.8	nsd	RL11 family; membrane glycoprotein
UL17	E	nsd	nsd	−24.6	nsd	
UL22A		†	nsd	−8.2	nsd	Secreted RANTES-binding protein
UL25	L	*	†	nsd	−5.9	UL25 family; tegument phosphoprotein
UL41A		†	nsd	−3.2	nsd	Putative membrane protein
UL44	E-L	†	nsd	−2.6	nsd	Processivity subunit of DNA polymerase
UL49	E-L	nsd	−7.3	nsd	−2.9	
UL80/UL80.5	L	*	‡	−14.1	nsd	Protease and capsid scaffold proteins
UL82	E-L	nsd	−2.9	−15.7	−5.7	DURP family; tegument phosphoprotein pp71; transcriptional activator; targeted to ND10 domains; targets Rb proteins for proteosomal degradation
UL83	E-L	†	†	−41.6	−5.4	DURP family; tegument phosphoprotein pp65; suppresses interferon response
**UL85**	E-L	*	‡	−19.6	nsd	Component of intercapsomeric triplexes in capsids
**UL86**	E-L	*	*	−14.1	nsd	Major capsid protein; component of hexons and pentons
UL89	E-L	*	‡	−5.0	nsd	Putative ATPase subunit of terminase
UL91	L	†	nsd	−4.9	nsd	
**UL94**	L	*	‡	−14.7	nsd	Tegument protein; binds ssDNA
**UL97**	E-L	†	nsd	−4.2	nsd	Serine-threonine protein kinase; phosphorylates UL44
**UL98**	E-L	nsd	−10.5	−12.1	−3.9	DNase
**UL99**	L	nsd	nsd	−8.8	−3.5	Myristylated tegument protein pp28
UL104	E	*	*	‡	−4.4	Capsid portal protein
UL112	E	nsd	nsd	−4.3	nsd	Orchestrates DNA replication proteins at viral replication compartments
**UL114**	E	*	†	−3.3	nsd	Uracil-DNA glycosylase
**UL115**	L	†	−2.0	−3.9	nsd	Virion envelope glycoprotein gL
**UL117**	E	†	nsd	−9.6	nsd	Required for viral replication compartments
UL119	E-L	*	nsd	−3.0	nsd	IgG Fc-binding membrane glycoprotein related to OX-2
UL122	IE-L	†	nsd	−36.6	−6.1	IE transcriptional activator IE2; interacts with basal transcriptional machinery and cellular transcription factors; specific DNA-binding protein
UL132		nsd	−3.3	−4.4	nsd	Virion glycoprotein
UL148		−3.6	nsd	−4.5	nsd	Putative membrane glycoprotein
**UL147A**		−3.1	nsd	−10.8	−4.2	Putative membrane protein
**UL146**		*	*	*	−34.4	CXCL family; putative secreted CXC chemokine
UL144		†	nsd	−8.8	nsd	Putative membrane glycoprotein; TNF-receptor homologue
UL141		*	nsd	−3.8	nsd	UL14 family; membrane glycoprotein; inhibits NK cell cytotoxicity by downregulating CD155
UL139		*	*	‡	−57.1	Putative membrane glycoprotein
IRS1	IE	−4.2	−7.4	nsd	nsd	US22 family, IE transcriptional activator, tegument protein, involved in host cell shut-off
**U373MG**						
RL11	E-L	†	4.9	nsd	sd	RL11 family; IgG Fc-binding membrane glycoprotein
RL13	E-L	†	2.2	nsd	nsd	RL11 family, virion envelope glycoprotein
UL4	E	nsd	−3.1	−3.6	nsd	RL11 family; virion envelope glycoprotein
UL10		†	†	2.5	nsd	RL11 family; putative membrane glycoprotein
UL11	E	nsd	nsd	3.9	nsd	RL11 family; membrane glycoprotein
UL13	E	nsd	nsd	2.8	nsd	Putative secreted protein
UL19		†	†	18.7	nsd	
UL21A		†	3.1	44.1	nsd	Facilitates viral DNA synthesis and late accumulation of IE transcripts
UL22A		†	nsd	−2.8	nsd	Secreted RANTES-binding protein
UL25	E	†	†	4	−4.6	UL25 family; tegument phosphoprotein
UL26		†	†	21.9	nsd	US22 family; tegument transactivator of MIEP
UL32	E	†	†	nsd	−4.1	Major tegument phosphoglycoprotein pp150; binds to capsids
UL33	E	†	†	25.1	nsd	GCPR family; virion protein; putative chemokine receptor
UL34		†	nsd	5.7	nsd	Repressor of US3 transcription
UL38	IE-E	†	†	6.1	nsd	Anti-apoptotic protein; protects cells from ER stress induced by unfolded protein response
UL43	L	†	nsd	3.7	nsd	US22 family; tegument protein
UL49	E-L	nsd	nsd	nsd	−2.2	
UL50		†	2.5	7.5	nsd	Inner nuclear membrane protein; facilitates capsid egress from nucleus
UL55	E-L	†	3.3	nsd	nsd	Viral envelope glycoprotein gB; mediates membrane fusion during entry
UL69	E-L	†	nsd	5.4	nsd	Regulatory protein; tegument protein; contributes to cell cycle block; involved in mRNA export; exhibits nucleocytoplasmic shuttling
UL82	E-L	nsd	nsd	nsd	−8.8	DURP family; tegument phosphoprotein pp71; transcriptional activator; targeted to ND10 domains; targets Rb proteins for proteosomal degradation
UL83	E-L	†	†	nsd	−8.4	DURP family; tegument phosphoprotein pp65; suppresses interferon response
UL85	E-L	†	nsd	−2.2	nsd	Component of intercapsomeric triplexes in capsids
**UL96**	E-L	†	nsd	2.8	nsd	Tegument protein
**UL98**	E-L	nsd	nsd	nsd	−4.7	DNase
**UL99**	L	nsd	nsd	nsd	−3.8	Myristylated tegument protein pp28
UL112	E	nsd	4.2	3	nsd	Orchestrates DNA replication proteins at viral replication compartments
**UL114**	E	†	†	2.8	nsd	Uracil-DNA glycosylase
**UL115**	L	†	2.1	nsd	nsd	Virion envelope glycoprotein gL
**UL116**	E-L	†	nsd	3.3	nsd	Putative membrane glycoprotein
**UL119**	E-L	†	nsd	4	nsd	IgG Fc-binding membrane glycoprotein related to OX-2
UL122	IE-L	†	nsd	nsd	−5.6	IE transcriptional activator IE2; interacts with basal transcriptional machinery and cellular transcription factors; specific DNA-binding protein
UL128	E-L	†	†	13.4	nsd	Viral envelope protein; cell tropism factor for endothelial, epithelial and leukocyte cells
**UL148**		nsd	2.8	nsd	nsd	Putative membrane glycoprotein
**UL147A**		nsd	5.3	3.4	−3.8	Putative membrane protein
**UL147**		nsd	6.7	8.1	−2.6	CXCL family; putative secreted CXC chemokine
**UL146**		†	†	†	−63.7	CXCL family; putative secreted CXC chemokine
UL145		†	†	13.1	nsd	RL1 family
UL140		†	†	nsd	−5.3	Putative membrane protein
UL139		†	†	nsd	−15.5	Putative membrane glycoprotein
**UL138**		nsd	nsd	2.6	nsd	Regulator of viral latency
**UL136**		†	nsd	3.5	nsd	Putative membrane protein
**UL135**		nsd	nsd	4.3	nsd	Putative secreted protein
US12	E	nsd	nsd	4.3	nsd	US12 family; putative multiple transmembrane protein
US13	E	nsd	nsd	6.4	nsd	US12 family; putative multiple transmembrane protein
US16	E	†	nsd	8.6	nsd	US12 family; putative multiple transmembrane protein
**US19**	E	nsd	nsd	5.7	nsd	US12 family; putative multiple transmembrane protein
**US20**	E	nsd	2.9	3.9	nsd	US12 family; putative multiple transmembrane protein

* Signal below detectable limits in HFFF-2s, RPE-1 and U373MGs.

† Signal not detected in HFFF-2s but detected in RPE-1s/U373MGs.

‡ Signal detected in HFFF-2s but not RPE-1s/U373MGs.

Differentially expressed genes were distributed among all temporal kinetic classes (Table 2). Twelve ORFs (UL44, UL80, UL85, UL86, UL89, UL94, UL97, UL98, UL99, UL104, UL114 and UL115) in RPE-1s and eight ORFs (UL50, UL55, UL69, UL85, UL98, UL99, UL114 and UL115) in U373MGs are responsible for conserved aspects of the replication cycle, in that they (core genes) were apparently inherited from the ancestor of all members of the family *Herpesviridae*. Among the families of HCMV genes (numbering 15) containing related members, the RL11 family (14 members) is strongly represented in the differentially expressed category: nine members in RPE-1s and five in U373MGs. In contrast, very few genes in the US22 family (12 members) were differentially expressed: one in RPE-1s and two in U373MGs. Five members of the US12 family (10 members) exhibited enhanced expression in U373MGs.

### Concordance between microarray and Northern blotting data

**Fig. 4.**
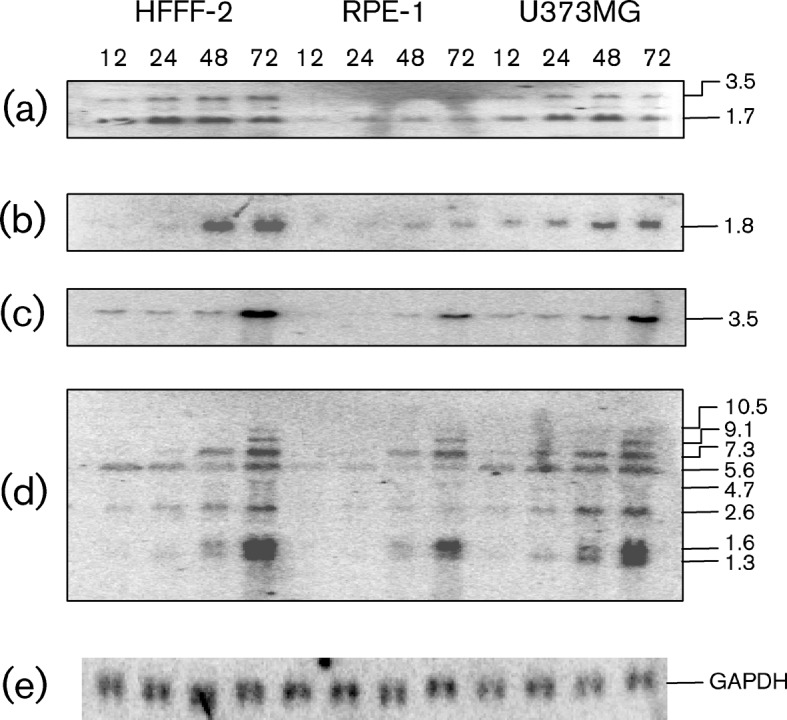
Northern blots showing representative HCMV transcripts produced in HFFF-2s, RPE-1s and U373MGs at 12, 24, 48 and 72 h p.i. (a) IRS1 (3.5 and 1.7 kb), (b) UL4 (1.8 kb), (c) UL83 (3.5 kb), (d) UL93 (10.5 kb), UL94 (9.1 kb), UL95 (7.3 kb), UL96 (5.6 kb), UL97 (4.7 kb), UL98 (2.6 kb) and UL99 (1.6 and 1.3 kb) and (e) glyceraldehyde 3-phosphate dehydrogenase (GAPDH). Genome co-ordinates (GenBank accession no. AY446894.2) of the HCMV probe sequences were: IRS1, 198 271–197 985; UL4, 13 936–142 429; UL83, 121 565–121 800; and UL99 145 796–146 085.

A selection of genes identified as differentially expressed in the microarray analysis were also assessed by Northern blotting (Fig. 4).

IRS1 and TRS1 span the junctions between U_S_ and TR_S_/IR_S_, and thus share identical promoters and 5′-coding sequences, but have different 3′-coding sequences (Chee *et al.*, 1990). Two IRS1-related RNAs were capable of binding the IRS1 microarray probe: a 3.5 kb full-length transcript and a 1.7 kb transcript initiated within the ORF. The protein encoded by the latter has been shown to antagonize expression of that encoded by the former (Romanowski & Shenk, 1997). In Northern blots, levels of both transcripts were significantly reduced at all time points in RPE-1s compared with HFFF-2s and U373MGs (Fig. 4), thus confirming the results of the microarray analysis.

UL4 produces a 1.8 kb transcript (Alderete *et al.*, 2001). In Northern blots, this transcript was produced in reduced amounts in RPE-1s throughout infection and in U373MGs at 48 and 72 h p.i., compared with HFFF-2s, thus confirming the results of the microarray analysis.

UL82 and UL83 are expressed as a 3.5 kb bicistronic transcript terminating downstream from UL82 (Dal Monte *et al.*, 1996). In Northern blots, the transcript level was similar in HFFF-2s and U373MGs to 48 h p.i., but moderately decreased (to 79 % of the level in HFFF-2s) at 72 h p.i. in U373MGs. In RPE-1s, the transcript was less abundant throughout infection than in HFFF-2s, reaching 54 % at 72 h p.i. These findings confirmed the results of the microarray analysis.

UL93, UL94, UL95, UL96, UL97, UL98 and UL99 are expressed as a 3′-co-terminal group of RNAs, which terminate downstream from UL99 and have L, L, E-L, E-L, E, E-L and L kinetics, respectively (Adam *et al.*, 1995; Wing & Huang, 1995). In Northern blots, UL93 (10.5 kb transcript) and UL95 (7.3 kb) were expressed at similar levels in all three cell lines. UL94 (9.1 kb), UL96 (5.6 kb), UL97 (4.7 kb), UL98 (2.6 kb) and UL99 (1.6 and 1.3 kb) were expressed at lower levels at 48 h p.i. in RPE-1s than in HFFF-2s. UL96 (5.6 kb) and UL98 (2.6 kb) were expressed at moderately higher levels in U373MGs than in HFFF-2s, and UL99 (1.6 and 1.3 kb) was produced in similar amounts in U373MG and HFFF-2s. These findings confirmed the results of the microarray analysis in regard to lower levels of expression of UL94, UL97, UL98 and UL99 in RPE-1s and higher expression of UL96 in U373MGs. However, results obtained for UL96 in RPE-1s and UL98 and UL99 in U373MGs differed from those obtained using the microarray.

## Discussion

Growth of HCMV strain Merlin was characterized in HFFF-2s, RPE-1s and U373MGs, which represent human fibroblast, epithelial and astrocyte cell types, respectively. Cell type-dependent differences were observed in viral CPE, infectious viral yields (CAV and CRV) and viral genomic yields. Viral growth in RPE-1s and U373MGs was characterized by delayed and reduced yields of CAV and CRV, relative to HFFF-2s (Fig. 2). Delayed viral growth kinetics have also been reported for primary RPEs infected with HCMV strain AD169 (Detrick *et al.*, 1996).

To investigate HCMV cell type-dependent transcriptional activity, a bespoke strain Merlin microarray was developed. Differential expression exhibited by 10 specimen genes was validated by Northern blotting. Minor discrepancies between the two approaches may be caused by several factors, including the higher sensitivity and statistical robustness of microarrays compared with Northern blots and the possible inability of microarray probes to discriminate fully among 3′-co-terminal transcripts (Table 2; Chambers *et al.*, 1999; Kennedy *et al.*, 2005; Stingley *et al.*, 2000). A total of 41 HCMV ORFs in RPE-1s and 48 ORFs in U373MGs were differentially expressed, compared with HFFF-2s. All differentially regulated ORFs in RPE-1s were expressed at lower levels, whereas some ORFs in U373MGs were expressed at higher levels and others at lower levels. The consequences of increased gene expression are unclear, but reduced expression of genes encoding regulatory or enzymic functions may play its part in restricting viral replication.

Gross differences in viral entry, initiation of gene expression or transition from E to L gene expression were ruled out as being responsible for differential expression of viral genes in RPE-1s and U373MGs, compared with HFFF-2s. The overall kinetics of gene expression were similar in RPE-1s and HFFF-2s, with expression of many ORFs being detected earlier in RPE-1s, and especially in U373MGs, compared with HFFF-2s (Table S1). Moreover, differential expression was not confined to genes of a particular temporal expression class. Despite viral DNA yields in RPE-1s and U373MGs being lower than in HFFF-2s, they appeared to be sufficient to maintain normal levels of transcription of most genes, including L genes.

### Functions associated with differentially expressed genes

To shed light on the mechanisms by which viral growth in RPE-1s and U373MGs is suppressed, differentially expressed genes were grouped according to functional involvement at landmark stages in the viral replication cycle (Table 3). At each stage in RPE-1s and U373MGs, expression of multiple viral genes was affected, thus demonstrating the broad effects of the underlying processes (Table 2). Downregulation of certain herpesvirus core genes in RPE-1s is compatible with a trend to temperance of virus replication. The situation is less clear for U373MGs, as differences in the levels of gene expression were generally smaller and many ORFs were upregulated, making the consequences for viral replication less predictable. However, most genes downregulated in U373MGs have roles in viral maturation and release. Many more replication steps were potentially impaired in RPE-1s than in U373MGs (Table 3), and yet higher infectious viral yields were generated (Fig. 2), implying a secondary obstacle to viral replication in U373MGs that has a greater impact on viral growth than impaired maturation. The apparent deregulation of the viral transcription cascade may be an important factor, likely to perturb post-transcripional events, including translation, post-translational processing, intracellular translocation of viral proteins and viral DNA synthesis, and thus contributing to the low infectious viral yields. In summary, a series of potential bottleneck stages (Table 3) that may impede, but not halt, progression through the viral replication cycle was proposed in RPE-1s and U373MGs. This may account for the characteristic cell type-dependent viral growth kinetics observed.

**Table 3.** Differentially expressed HCMV genes grouped according to functional class in the replication cycle Expression is given in relation to HFFF-2s. No gene in these classes was overexpressed in RPE-1s.

Functional class	RPE-1 underexpressed	U373MG overexpressed	U373MG underexpressed
Regulation of gene expression	IRS1	UL26	UL122
	UL122	UL69	UL82
	UL82		
Development of replication compartments	UL112	UL112	
	UL117	UL114	
	UL114		
DNA replication and processing	UL44	UL114	UL98
	UL89	UL21A	
	UL97		
	UL98		
	UL114		
Capsid assembly and genome encapsidation	UL80		UL85
	UL85		UL98
	UL86		
	UL97		
	UL98		
	UL104		
Maturation and release	UL4	UL25 (48 h p.i.)	UL25 (72 h p.i.)
	UL25	UL26	UL4
	UL82	UL43	UL32
	UL83	UL50	UL82
	UL94	UL55	UL83
	UL97	UL69	UL99
	UL99	UL96	
	UL115	UL115	
		UL128	
Immune evasion	RL11	RL11	
	UL119	UL22A	
	UL141	UL33	
	UL144	UL119	

HCMV transcriptome expression was investigated over the period 12–72 h p.i., during which there was a 24 h delay in production of CAV in both RPE-1s and U373MGs, compared with HFFF-2s. In RPE-1s, and to a lesser extent in U373MGs, CAV yield increased at 72 h and 96 h p.i. (Fig. 2a). It is possible that, as the protein products of underexpressed genes reach a critical level, replication bottlenecks are cleared sequentially. Moreover, accumulation of CAV in U373MGs was much less than that in RPE-1s, and the burst of intracellular infectivity in both cell lines at 72 h p.i. remained largely cell-associated thereafter. Thus, it appears that the bottlenecks affecting CRV yield remained largely in force for U373MGs, perhaps because the cognate functions involved in virion maturation and release were provided by viral structural proteins that are needed in high abundance.

It is notable that HCMV genes specifying immune evasion functions were differentially regulated in RPE-1s and U373MGs (Table 3). Although immune evasion genes affect viral pathogenesis *in vivo*, the effects of downregulation on viral replication in cell culture are unknown.

### Cell type-specific factors that may modulate HCMV gene expression

Cell type-dependent differences in the interactions between host and virus are probably responsible for modulation of HCMV gene expression. The eye, and to a lesser extent the brain, are immune privileged organs, and their response to pathogens differs from that of other tissues and organs. The RPE layer is part of the blood–retina barrier and plays an important part in immune privilege (Holtkamp *et al.*, 2001). The immune response of the eye to HCMV infection is weak, usually lacks neutrophil involvement, and results in persistent infection of retinal tissue (Cinatl *et al.*, 2000) and low levels of viral replication (Scholz *et al.*, 2003). The observed minimal CPE in RPE-1s and maintenance of cell-to-cell contact in the monolayer are consistent with preservation of the retinal barrier. Immune privilege in the eye may also explain the downregulation of some HCMV immune evasion genes in RPE-1s.

Binding of the HCMV envelope glycoproteins gB and gH to receptors on fibroblasts, and most other cell types, causes rapid induction of NF-κB activity, resulting in activation of the viral major immediate-early promoter (MIEP) and cellular genes that function in cell-mediated inflammatory responses, including those involving cyclooxygenase-2 (COX-2) and interferons (Eickhoff & Cotten, 2005). However, the NF-κB pathway and COX-2 are not induced in HCMV-infected RPE cells (Cinatl *et al.*, 2001; Scholz *et al.*, 2003). COX-2 mediates the synthesis of prostaglandins, which are involved in acute and chronic inflammatory and innate immune responses. Thus, avoidance of NF-κB activation in RPE cells would circumvent prostaglandin-associated inflammatory responses in the eye and limit viral replication in the retina (Scholz *et al.*, 2003). Inhibition of COX-2 activity in HCMV-infected fibroblasts results in reductions in viral mRNA levels, viral DNA synthesis, viral growth and cell-to-cell spread of infectivity (Schröer & Shenk, 2008; Zhu *et al.*, 2002). Thus, low levels of COX-2 in RPE-1s may contribute to impaired viral replication.

An investigation of HCMV infection in the brain showed that neoplastic astroglial cells are permissive for HCMV, while non-neoplastic cells are not (Poland *et al.*, 1990). The ability of HCMV to grow in neoplastic astrocytomas is considered to be dependent upon the degree of cellular differentiation, suggesting that differentiation-dependent cellular genes may control the HCMV transcriptional cascade. HCMV strain Merlin developed a CPE in U373MGs that was characterized by large numbers of swollen and syncytial cells. Nevertheless, cell-to-cell contact appeared to be maintained, suggesting the preservation of a barrier cell layer, as in the case of RPE-1s. Infectious viral yields from U373MGs were very low compared with those from HFFF-2s and RPE-1s, and viral genomic yield was 129-fold lower than in infected HFFF-2s at 96 h p.i. Lower expression levels of genes with roles in viral maturation and release of infectious progeny identify these stages as probable bottlenecks (Table 3). However, the apparent deregulation of the HCMV transcription cascade in U373MGs probably accounts for the greater part of the effects on viral yields and growth kinetics.

HCMV utilizes many cellular transcription factors to modulate viral gene expression, and cell-type differences in the repertoire and levels of these factors may impact viral transcriptome activity. The MIEP has multiple binding sites for a wide range of factors (Stinski & Meier, 2007), binding sites for certain factors have been identified in UL4 (Chen & Stinski, 2000), UL98 (Adam *et al.*, 1995) and UL112 (Rodems *et al.*, 1998), and many other HMCV genes are associated with p53-binding sites (Hannemann *et al.*, 2009). In HCMV-infected cells, p53 is sequestered in viral replication compartments in the nucleus (Fortunato & Spector, 1998; Rosenke *et al.*, 2006) and modulates the expression of at least one HCMV gene (UL94) (Casavant *et al.*, 2006; Wing *et al.*, 1998). The HCMV genome contains 21 consensus-binding sites for p53, located either in the promoters or coding sequences of genes that have roles in gene regulation, DNA replication and packaging, and maturation and envelopment of viral particles (Rosenke *et al.*, 2006). U373MGs have a mutation (R273H) in p53 (Van Meir *et al.*, 1994). Studies comparing HCMV gene expression in fibroblasts either expressing or not expressing p53 led to the identification of 22 differentially expressed genes, 11 of which were downregulated in non-expressing cells, resulting in delayed and reduced infectious viral yields (Hannemann *et al.*, 2009). Of the HCMV genes that were differentially expressed in RPE-1s or U373MGs, 17 contain potential p53-binding sites (Hannemann *et al.*, 2009; Rosenke *et al.*, 2006) and may respond to cell type-dependent differences in p53 activity. The unusually rapid expression of viral E and L genes in U373MGs may be a consequence of the mutated p53 protein. In transient transfection studies, the mutated p53 protein activated the HCMV MIEP, and the additional presence of CREB enhanced this activity. Since p53 mutant-mediated transactivation requires only a functional TATA box (Deb *et al.*, 1992), it is possible that viral promoters are activated non-specifically in U373MGs, regardless of their normal temporal kinetics.

HCMV has evolved the capacity to replicate in various cell types that differ in expressed cellular genes and perform specialized functions in the context of particular organs or tissues. It remains to be determined whether the transcriptome activity of strain Merlin in HFFF-2s, RPE-1s and U373MGs is mirrored in the cognate cell types *in vivo*. We have shown that expression of particular HCMV genes in cultured cells is modulated in response to different intracellular environments. Our data suggest that the imperatives of immune privilege in RPE-1s and U373MGs and differences in expressed cellular factors result in reduced transcription from multiple viral genes. However, it is highly probable that additional cell type-dependent factors not identified here also contribute to cell type-specific differences in viral growth kinetics, including differential translation of transcripts, post-translational processing and intracellular translocation of viral products.

## Methods

### 

#### Virus and cell lines.

HCMV strain Merlin (Dolan *et al.*, 2004; RL13^–^UL128^–^) was used. Monolayers of HFFF-2s (ECACC; 86031405) and U373MGs (ECACC; 89081403) were grown in Dulbecco’s modified Eagle’s medium (DMEM) supplemented with 10 % FCS. hTERT-immortalized RPE-1s (C4000-1; Clontech) were grown in DMEM/Ham’s F-12 supplement containing 10 % FCS.

#### Susceptibility to infection.

HFFF-2s, RPE-1s or U373MGs (2×10^5^ cells) were infected with HCMV at 6 p.f.u. per cell, and the number of infected cells in two fields of view (nominally 500–600 cells per field) was estimated by immunofluorescence at 48 h p.i. by using an UL44 antibody (CCH2-FITC; Dako).

#### Determination of viral genome yields.

HFFF-2s, RPE-1s and U373MGs (4×10^5^ cells) were infected with HCMV at 6 p.f.u. per cell, and whole cell DNA was harvested at 24, 48, 72 and 96 h p.i. DNA was also harvested at 48 h from MI cells. The monolayers were trypsinized and cells were pelleted at 550 ***g*** for 10 min at room temperature. Infected cell DNA extracts were prepared by using a FlexiGene kit (Qiagen). Viral genome yield was estimated by qPCR using UL130 gene primers (5′-GCGAGGGATAGAGAAAAGGACAG-3′ and 5′-CCGTGGTCGACGCTAACAG-3′) and a TaqMan probe (5′-6-FAM-CGGTTTGGAATACGTCAGT-MGB-3′). Each experiment involved amplification of MI, infected cell and control plasmid DNAs, with four reactions per sample. The plasmid, which contained the UL130 ORF, was used to generate a standard curve for determination of HCMV genome copy number in infected cell samples. Samples were amplified (40 cycles at 95 °C for 3 s followed by 60 °C for 30 s) in an Applied Biosystems 7500 Fast Real-time PCR System, and data were analysed using proprietary software.

#### Preparation of RNA from MI and HCMV-infected cells.

For microarray analysis, HFFF-2s, RPE-1s and U373MGs were infected simultaneously with a single HCMV preparation, and RNA was harvested at 12, 24, 48 and 72 h p.i. For each cell line, 12 monolayers (each 6×10^6^ cells) were infected at 6 p.f.u. per cell, thus providing three biological replicates for each time point. MI cultures were harvested at 72 h p.i. Cells were solubilized in lysis buffer containing 1 % 2-mercaptoethanol, and total cell RNA was extracted using an RNeasy kit (Qiagen). After treatment with DNase I (Invitrogen), the quality and purity of RNA preparations were confirmed by lack of smearing of the 28S and 18S rRNA bands after electrophoresis on 1 % agarose/2.2 M formaldehyde gels. Elimination of DNA from the preparations was confirmed by failure to amplify a cellular DNA sequence (from the lactate dehydrogenase gene) by PCR.

#### Northern blot analysis.

RNA (10 µg per track) was loaded onto a 1 % agarose/2.2 M formaldehyde gel, separated by electrophoresis, transferred by blotting to Hybond-N+ membrane (Amersham Biosciences), and fixed by irradiation in a UV-cross-linker (Stratagene) at 12 000 J cm^−2^. DNA probes were prepared from PCR-generated templates by using a Rediprime II random prime labelling kit (Amersham Biosciences), with direct incorporation of [α-^32^P]dCTP (50 µCi) (Amersham Biosciences), diluted and denatured in Rapid-hyb buffer (Amersham Biosciences), and hybridized to the membrane overnight at 68 °C. The hybridized membranes were washed and labelled RNA bands were visualized by using a Bio-Rad Personal FX phosphorimager.

#### Microarray probe design and fabrication.

Specific 60-mer oligonucleotide probes (Table S2) were generated for HCMV ORFs (GenBank accession no. AY446894.2) according to the protocol published previously (Ebrahimi *et al.*, 2003), synthesized by MWG-Biotech (USA), and printed in triplicate on Corning GAPS II microscope slides, with 16 copies of the microarray on each slide. Probes for two *Bacillus subtilis* genes (*spoOB*, GenBank accession no. M24537, and *lys*, GenBank accession no. X17013) were utilized in both sense and antisense orientations in order to assess array specificity and sensitivity and to serve as normalization controls. The array was validated by hybridization against MI and infected cell cDNAs at a range of temperatures in the presence or absence of spiked controls.

#### Synthesis of cDNA for microarray hybridization.

RNA aliquots (25 µg) were spiked with *B. subtilis* control mRNAs (50 ng each), mixed with 1 µg random hexamer primers (Invitrogen), incubated at 70 °C for 10 min, and cooled on ice. Labelled cDNAs were then produced by incubating the samples at 42 °C for 2 h with Cy3-dCTP (Roche), dNTPs (Roche) and Superscript II reverse transcriptase (Invitrogen).

#### Microarray hybridization, data collection and data processing.

Labelled cDNAs were prepared for hybridization as described previously (Ebrahimi *et al.*, 2003). Hybridization was performed in a humidified chamber (Genetix). Array specificity was confirmed by hybridization with cDNAs prepared from MI- or HCMV-infected cell RNAs. Arrays were scanned at 543 nm with a pixel resolution of 10 µm using ScanArray Express hardware and associated software (Perkin-Elmer). The background intensity value was subtracted from the corresponding spot intensity, and the mean value for each probe was calculated (*n* = 3). The linear dynamic range was determined by using published methods (Ebrahimi *et al.*, 2003).

The signal threshold that gave a false positive rate of 5 % was determined from receiver operating characteristic analysis of the control data. Datasets were then scaled against the 75th percentile of the positive-control signal. Probes with signal values falling below the signal threshold cut-off point were assigned a signal value of 0.01 to facilitate log-transformation and further analysis of data. ORFs UL48A, US1 and US34A gave signal values below the detection limits of the assay for all time points in each cell line, and were not analysed further.

Array variability was checked by measuring the median signal intensity for a given cell line and time point p.i. on log-transformed data. Only arrays where total median signal intensity values were within threefold of each other were selected for further analysis. To identify differentially expressed genes, mean log_2_ signal intensity values were calculated for all viral ORFs at each time point in the three cell lines. Where values were >0.01 in both the control (HFFF-2s) and test cells (RPE-1s and U373MGs), signal intensity fold change (FC) values were computed. The probability of cell type-dependent differential gene expression (*P<*0.05) was then determined by Student’s *t*-tests of FC values for each time point (Table S1).
